# Effect of intrinsic membrane conductances on Phase Resetting Curves in a conductance-based neuron model

**DOI:** 10.1186/1471-2202-13-S1-P68

**Published:** 2012-07-16

**Authors:** Wafa Soofi, Astrid Prinz

**Affiliations:** 1 Department of Biomedical Engineering, Georgia Institute of Technology/Emory University, Atlanta, GA, 30332, USA; 2 Department of Biology, Emory University, Atlanta, GA, 30322, USA

## 

It is well known that the magnitude and dynamics of a neuron’s membrane conductances influence the nature of its response to synaptic input [1]. However, the effect of specific membrane conductance values on the neuron’s response to perturbations (such as inhibitory synaptic input) has not yet been rigorously examined. In this work, we use a conductance-based model of a spontaneously bursting pyloric neuron of the crustacean stomatogastric ganglion (STG) to examine the relationship between intrinsic membrane conductances and neuronal output in response to perturbations. We describe the effect of these perturbations using a Phase Resetting Curve (PRC), which indicates the magnitude of the delay or advancement of a periodically bursting neuron’s trajectory along its limit cycle as a function of the perturbation’s timing [2]. The present study utilizes an existing database of single-compartment conductance-based model neurons [3] to determine the effects of specific membrane conductances on the shape of the PRC. The database consists of about 1.7 million model neurons, each of which was obtained by independently varying eight maximal conductances over six equally spaced possible values. PRCs for each of the bursting model neurons were obtained by simulating 1000 nS of inhibitory synaptic input at different phases of the rhythmic activity of each model neuron and measuring the resulting period change relative to the free-running burst period [3]. For all regularly bursting neurons with analyzable PRCs, we examined the effect of systematically varying each conductance upon specific features of the PRC, including the minimum delay, maximum delay, and neutral phase point. Increasing the leak conductance resulted in a decrease in magnitude of the minimum and maximum values of the PRC, indicating that the PRC was shifted toward smaller delays. We further saw that certain conductance pairs, including the hyperpolarization-activated and leak conductances, exhibit a correlation in model neuron populations with tightly constrained PRC attributes, suggesting that this conductance pair may aid in preserving PRC shape. Preliminary results also suggest that the effects of certain membrane conductances upon PRC shape are influenced by the magnitude of the synaptic input.
